# Serum exosomes lncRNAs: TCONS_I2_00013502 and ENST00000363624 are new diagnostic markers for rheumatoid arthritis

**DOI:** 10.3389/fimmu.2024.1419683

**Published:** 2024-07-09

**Authors:** Han Wu, Qiuhua Chen, Sijie Wang, Chunlong Yang, Li Xu, Haiyan Xiao, Tong Xie, Qingjun Pan

**Affiliations:** ^1^ Clinical Research and Experimental Center, Affiliated Hospital of Guangdong Medical University, Zhanjiang, Guangdong, China; ^2^ Clinical Laboratory, The Second Affiliated Hospital of Guangdong Medical University, Zhanjiang, Guangdong, China; ^3^ Department of Immunology and Rheumatology, Affiliated Hospital of Guangdong Medical University, Zhanjiang, Guangdong, China; ^4^ Department of Cellular Biology and Anatomy, James and Jean Culver Vision Discovery Institute, Medical College of Georgia, Augusta University, Augusta, GA, United States; ^5^ Guangdong Provincial Key Laboratory of Autophagy and Major Chronic Non-communicable Diseases, Affiliated Hospital of Guangdong Medical University, Zhanjiang, China

**Keywords:** rheumatoid arthritis, serum exosomes, long non-coding RNA, diagnose, biomarker

## Abstract

The lack of diagnostic markers limits the window of effectiveness for rheumatoid arthritis (RA) therapies. Here, we isolated exosomes of serum samples from four distinct groups RA patients, according to disease activity and with/without medication. Then, total RNA of exosomes was extracted for whole-transcriptome sequencing. Focusing on lncRNA sequencing, gene ontology (GO) and kyoto encyclopedia of genes and genomes (KEGG) pathway enrichment analyses were performed. We found that the number of upregulated lncRNAs were significantly higher than that of downregulated lncRNAs in each four RA groups. And most importantly, we identified two specific lncRNAs from differentially expressed lncRNAs, TCONS_I2_00013502 (up-regulated) and ENST00000363624 (down-regulated) in RA. Receiver Operating Characteristic (ROC) curve analysis showed that the two lncRNAs were promising biomarkers for RA diagnosis. These findings highlight lncRNAs of the serum exosome are important biomarkers and provide application potential for diagnosis of RA.

## 1 Introduction

Rheumatoid arthritis (RA) is a chronic autoimmune disease characterized by joint inflammation and bone damage caused by complex pathogenic factors (1, 2). The pathogenesis of RA is complex, involving pro-inflammatory factors, such as Interleukin-1, Interleukin-17, Interleukin-22, TNF-α, Interleukin-6, and MMP (3–5). However, the specific mechanisms remain unclear. While diagnostic methods for RA are limited and diagnosis often delayed, a considerable number of RA patients endure ineffective relief. Hence, comprehensive research on RA’s molecular mechanisms and the discovery of swift, novel diagnostic markers are critical in preventing and mitigating the onset and progression of RA, reducing patient distress.

Exosomes are extracellular vesicles formed by the inward budding of the cell membrane to create endosomes, which form multivesicular bodies before being secreted into the extracellular space. Their diameters range from 30 to 150 nm (6). Almost all cell types release exosomes that are present in various body fluids, including urine, blood plasma, saliva, cerebrospinal fluid, neural fluid, and breast milk etc. The double-membrane structure protects the exosomes, allowing them to provide stable multi-omic information (7). Current research has found that exosomes are rich in nucleic acids (long non-coding RNA (lncRNA), microRNA, circRNA, mRNA, tRNA, etc.), proteins, cholesterol, and other bioactive substances (8). After entering the external environment, exosomes are absorbed by cells through autocrine or paracrine pathways. They can also be absorbed and released by distant target tissues or organs through the circulatory system, participate in cell communication and substance transfer, and directly or indirectly regulate the functions and phenotypes of recipient cells (9). Studies have shown that lncRNAs are often enriched in exosomes and rely on exosome transport to exert their biological functions (10, 11), making them an increasingly important focus of attention.

In the human genome, only 2% of genes encode proteins (mRNA), and the remaining 98% produce non-coding RNAs, with those longer than 200 nucleotides classified as the lncRNA (12). Early research considered lncRNAs as by-products of RNA polymerase II transcription without biological functions and lacking the ability to encode proteins. However, lncRNAs have been found to participate in various crucial regulatory processes, including X chromosome silencing, chromatin modification, transcriptional activation and interference, and post-transcriptional modifications etc. (13). The regulatory roles of lncRNAs have garnered widespread attention. Some lncRNAs have been reported to encode various functional peptides or polypeptides that play specific biological roles. Abnormal expression and function of lncRNAs are closely associated with human diseases, including cancer, psychiatric disorders, and autoimmune diseases, among others (14, 15). Most studies have focused on evaluating the expression profiles of non-coding RNAs in synovial cells, tissues, or synovial fluids of patients with RA. In addition, obtaining samples from the joint cavity are limited that cause inconvenience in the diagnosis and treatment of RA (16). Therefore, it’s important for developing more convenient, rapid, and accurate diagnosis of RA and further explore the potential roles of lncRNAs in the pathogenesis of RA.

Here in this study, we collected peripheral blood serum samples from patients with RA and controls, extracted exosomes, performed RNA sequencing, and analyzed and compared the expression profiles of lncRNAs in exosomes from different groups. We screened and validated two specific lncRNAs, TCONS_I2_00013502 and ENST00000363624 in RA. The ROC analysis demonstrated that the combination of these two lncRNAs and anti-cyclic citrullinated peptide (anti-CCP) significantly improved the accuracy of RA diagnosis. These findings highlight the lncRNAs of serum exosome are important biomarkers and provide application potential for diagnosis of RA.

## 2 Methods

### 2.1 Patient and clinical data collection

Thirteen patients with RA, who were admitted to the Affiliated Hospital of Guangdong Medical University between December 2019 and December 2020, were included in this study. All enrolled patients were diagnosed with RA through clinical and relevant laboratory examinations, as well as imaging studies. The age range of the patients was 32–70 years. Additionally, five healthy volunteers aged 40–50 years were recruited as the normal group.

The study enrolled healthy individuals as participants, and there were no statistically significant differences in age or sex between the groups (*p* > 0.05). We classified all recruited patients into three groups: highly active patients without medication were labeled as RA-HW (DAS28 > 5.1), minimally active patients without medication were labeled as RA-LW (2.8 < DAS28 <5.1), and patients with improved conditions due to medication were labeled as RA-L (DAS28 < 3.2) (**Table 1**). Individuals with concurrent tumors, allergies, infections, or other autoimmune diseases were excluded.

**Table 1:** Characteristics of the patients and the normal group.

	Normal (n = 5)	RA-LW (n = 3)	RA-HW (n = 5)	RA-L (n = 5)
Age (years)	44.6 ± 8.3	56 ± 11.59	49.6 ± 4.86	56.4 ± 4.32
Gender (female/male)	3/2	3/0	5/0	3/2
Erythrocyte sedimentation (mm/h)	_	85 ± 16.44	101.6 ± 11.29	25.4 ± 5.77
Number of painful joints	_	1 ± 1	2.20 ± 0.86	0.2 ± 0.2
Number of swollen joints	_	2.67 ± 0.88	12.8 ± 3.22	1.2 ± 0.8
Rheumatoid factor	_	84.7 ± 16.26	1574 ± 859.3	30.66 ± 6.55
CRP	_	26.99 ± 22.16	32.22 ± 3.55	10.21 ± 7.92
Overall evaluation of doctorsc	_	0.57 ± 0.57	5.69 ± 0.14	1.52 ± 0.41
DAS28 score	_	3.42 ± 0.63	6.14 ± 0.68	2.71 ± 0.53

### 2.2 Ethical approval

This study was approved by the Ethics Committee of the Affiliated Hospital of Guangdong Medical University (PJ2014072). Written informed consent was obtained from all enrolled patients before participation. The study protocol complied with the Declaration of Helsinki.

### 2.3 Study design

In the exploratory phase, the expression profiles of lncRNAs in exosomes derived from the serum of 13 patients with RA and 5 healthy individuals were investigated through sequencing.

In the validation phase, differentially expressed lncRNAs were selected, and quantitative polymerase chain reaction (qPCR) was performed to validate the findings using serum samples from 32 patients with RA and 32 healthy controls. The concentration of anti-CCP were simultaneously measured by cobas-602. The anti-CCP test result is considered positive when the value exceeds 5 RU/ml.

### 2.4 RNA library construction, quality control, and sequencing

#### 2.4.1 Serum sample processing

The blood (10 ml) was collected by using a blood collection needle and a regular serum tube (without any agents). Allow it to sit at room temperature for 30 minutes, followed by undisturbed incubation at 4°C for 3–4 hours. Then, carefully transfer the pale yellow serum (approximately 4 ml) into a 15 ml tube and centrifuge at 3000 × g for 15 minutes at 4°C. Finally, transfer the collected serum into a separate cryotube and store it at -80°C.

#### 2.4.2 Extracellular vesicle extraction -Refer to exoEasy Maxi Kit (Qiagen) exoEasy Maxi Kit - Catalog no. 76064

a. Using pre-filtered serum, the supernatant was filtered to exclude particles larger than 0.8 μm.

b. Add 1 volume of buffer XBP to 1 volume of the sample and gently mix by inverting the tube 5 times. Allow the mixture to warm up to room temperature (22–28°C).

c. Add 16 ml of the sample/XBP mix onto the exoEasy spin column and centrifuge at 500 × g for 1 minute. Discard the flow-through and place the column back into the same collection tube. If the sample volume exceeds 8 ml, repeat this step until the entire volume has passed through the column.

d. Add 10 ml of buffer XWP and centrifuge at 5000 × g for 5 minutes to remove residual buffer from the column. Discard the flow-through along with the collection tube.

e. Transfer the spin column to a fresh collection tube.

f. Add 400 μl of buffer XE to the membrane and incubate for 1 minute. Centrifuge at 500 x g for 5 minutes to collect the eluate.

g. Re-apply the eluate to the exoEasy spin column membrane and incubate for 1 minute. Centrifuge at 5000 × g for 5 minutes to collect the eluate and transfer it to an appropriate tube.

#### 2.4.3 RNA extraction and quality control-Refer to invitrogen kit

a. Retrieve the extracellular vesicle sample from the -70°C freezer, thaw it, and centrifuge it at 4°C, 12,000 × g for 10 minutes to remove potential impurities.

b. Take 250 μl of extracellular vesicle fluid and transfer it to a 1.5 ml centrifuge tube. Add 750 μl of TRIzol LS Reagent and vigorously shake the tube manually to mix.

c. Centrifuge at 12,000 × g for 5 minutes and discard the precipitate.

d. Add chloroform (200 μl chloroform/ml Trizol) and mix well for 15 minutes. Keep it at room temperature for 15 minutes. Note: Avoid using a vortex mixer to prevent genomic DNA fragmentation.

e. Centrifuge at 4°C, 12,000 × g for 15 minutes.

f. Transfer the upper aqueous phase (1 ml Trizol - 400 μl) to another centrifuge tube.

g. Add NANA (0.5 ml NANA/ml Trizol) and mix well. Keep it at room temperature for 5–10 minutes.

h. Centrifuge at 4°C, 12,000 × g for 10 minutes, discard the supernatant, and allow the RNA to settle at the bottom of the tube.

i. Add 75% ethanol (1 ml 75% ethanol/ml Trizol) to the precipitate, gently shake, and centrifuge the tube to suspend and precipitate the RNA.

j. Centrifuge at 4°C, 8000 × g for 5 minutes.

k. Air-dry or vacuum dry it at room temperature for 5–10 minutes.

l. Dissolve the RNA sample in 50 μl H2O (Thermo Scientific, AM9932) at 55–60°C for 5–10 minutes. Store it for later use.

LncRNA library profiles were prepared following the manufacturer’s instructions. In detail, the Ribo-Zero rRNA Removal Kits (Illumina, USA) were used to remove rRNAs from the enriched total RNAs of serum exosomes. RNA was preprocessed using the TruSeq Stranded Total RNA Library Prep Kit (Illumina, USA) to construct sequencing libraries. Library quality control and quantification were performed using a Bioanalyzer 2100 instrument (Agilent Technologies, USA). According to Illumina sequencing protocols, the 10 pM library was denatured into single-stranded DNA molecules, captured on an Illumina flow cell, and subjected to *in situ* amplification to form clusters. An Illumina HiSeq 4000 sequencer was used for 150-cycle sequencing to generate paired-end reads. The cutadapt (v1.9.3) software was used to trim adapters, remove low-quality reads, and obtain high-quality reads.

Raw reads were obtained from an Illumina HiSeq sequencer after image recognition and base calling. The cutadapt software was then used to remove adapters, filter out low-quality reads, and obtain high-quality clean reads. The hisat 2 software aligned the clean reads to the human reference genome (UCSC HG19). Subsequently, guided by the gtf gene annotation file, the cuffdiff software (part of the cufflinks software suite) was used to obtain transcript-level fragments per kilobase of exon per million fragments mapped (FPKM) values for lncRNAs. These values were considered the expression profiles for lncRNAs, and fold changes and p-values between the two sample groups were calculated to identify the differentially expressed lncRNAs. Additionally, we conducted GO and KEGG pathway enrichment analyses using R clusterProfiler 4.11.1 for genes derived from differentially expressed lncRNAs.

### 2.5 Candidate lncRNAs selection

Three groups of commonly expressed lncRNAs were selected based on their fold change (|FC| > 1.5) and p-values (*p* < 0.05), resulting in the identification of upregulated and downregulated lncRNAs (**Table 2**).

**Table 2:** Differential expression of the lncRNAs in the exosomes.

Transcript_id	Gene ID	Loacation	LogFC	P value	Trend
TCONS_00026389	XLOC_012738	Chromosome 18	1.54752	0.00005	up
TCONS_00028428	XLOC_013779	Chromosome 20	2.92426	0.00035	up
TCONS_00028193	XLOC_013543	Chromosome 20	2.38456	0.00005	up
TCONS_00028189	XLOC_013543	Chromosome 20	2.92384	0.0078	up
TCONS_00028422	XLOC_013773	Chromosome 20	2.54799	0.00005	up
TCONS_00028426	XLOC_013778	Chromosome 20	2.6819	0.00005	up
TCONS_l2_00013502	XLOC_l2_007237	Chromosome 2	2.11607	0.00005	up
TCONS_l2_00003048	XLOC_l2_001591	Chromosome 10	2.64452	0.00005	up
NR_033191	PPP2R2D	Chromosome 10	3.18688	0.01485	up
uc010wia.1	AK027091	Chromosome 17	3.18205	0.00005	up
ENST00000363624	ENSG00000200494	Chromosome 20	1.58129	0.00005	down
ENST00000365328	ENSG00000202198	Chromosome 6	3.06096	0.00005	down
ENST00000363618	ENSG00000200488	Chromosome 2	-3.9837	0.0001	down
ENST00000458748	ENSG00000270066	Chromosome 1	2.45178	0.022	down
ENST00000437681	ENSG00000242125	Chromosome 1	2.92414	0.0201	down

### 2.6 RT-qPCR analysis

Total RNA of thirty-two serum samples from patients with RA were extracted using an exoRNeasy Maxi Kit (Invitrogen, USA). Their concentrations were measured using a NanoDrop spectrophotometer ND-1000 (Thermo Fisher, USA). The RNA from each sample was reverse-transcribed into cDNA using a QuantiNova Reverse Transcription Kit (Qiagen, Germany). Subsequently, the SYBR Green Kit (Takara, Japan) was used for qPCR to detect the expression levels of the lncRNAs. The PCR amplification program included an initial denaturation step at 95°C for 5 minutes, followed by 40 cycles at 95°C for 10 s and 60°C for 60 s. The 2^−ΔΔCt^ method was then used to calculate the expression levels of each lncRNA, with glyceraldehyde-3-phosphate dehydrogenase (GAPDH) serving as the reference gene. The expression of 18 candidate lncRNAs were calculated using the 2^-ΔΔCt^ method with the nematode cel-mir-39 as an external reference. The primer sequences are listed in **Table 3**.

**Table 3:** Primer sequences of the differentially expressed lncRNAs in the exosomes.

LncRNA	Forward (5’ to 3’)
TCONS_00026389	CAATGCTGTGTAGCCAGAGCCTAG
TCONS_00028428	ATCGTGAAACAGAAGACCCAGAAAGG
TCONS_00028193	GCTGCTGTTCAAATGGCTCCTTTC
TCONS_00028189	ATCGTGAAACAGAAGACCCAGAAAGG
TCONS_00028422	GCTGCTGCTGCTGTTCAAGTTTG
TCONS_00028426	TTTGGGTTGAGCCGCTGTTGTAG
TCONS_l2_00013502	CTTCCCCAATGCTTATGGAACG
TCONS_l2_00003048	AAGTAGTCTGTGATGGATGCGTGTTC
NR_033191	TCAGATACCGCTCTTTCTCCAACTTTC
uc010wia.1	GATTTGGACGAGAGACACAGGATGAG
ENST00000363624	GAGTGCAGTGGTGTTTACAACT
ENST00000365328	TAGAGGAGGACCGGTCTTCG
ENST00000363618	AGGACGACCATCCCCGATAG
ENST00000458748	CTTGGAGCGTGTTAGGCGAGTG
ENST00000437681	TGGGACTGAAGGGGGATCAT

### 2.7 Statistical methods

Statistical analyses were performed using software SPSS 26 and GraphPad Prism 9. The data in this study exhibited a non-normal distribution. To compare two variables with non-normally distributed metric data, the Wilcoxon test was applied. The Kruskal–Wallis test was used for comparisons involving multiple variables. Owing to the heterogeneity of variance, the dispersion was expressed as median (range) or quartiles. Receiver operating characteristic (ROC) curves were generated to assess the diagnostic value of differentially expressed genes in RA. A *p* values < 0.05 were considered statistically significant, the level of significance was set at *p* < 0.05 (*), *p* < 0.01 (**) or *p* < 0.001 (***).

## 3 Results

### 3.1 Analysis of the differentially expressed lncRNAs between the RA-HW group and the normal control group

**Figure 1:**
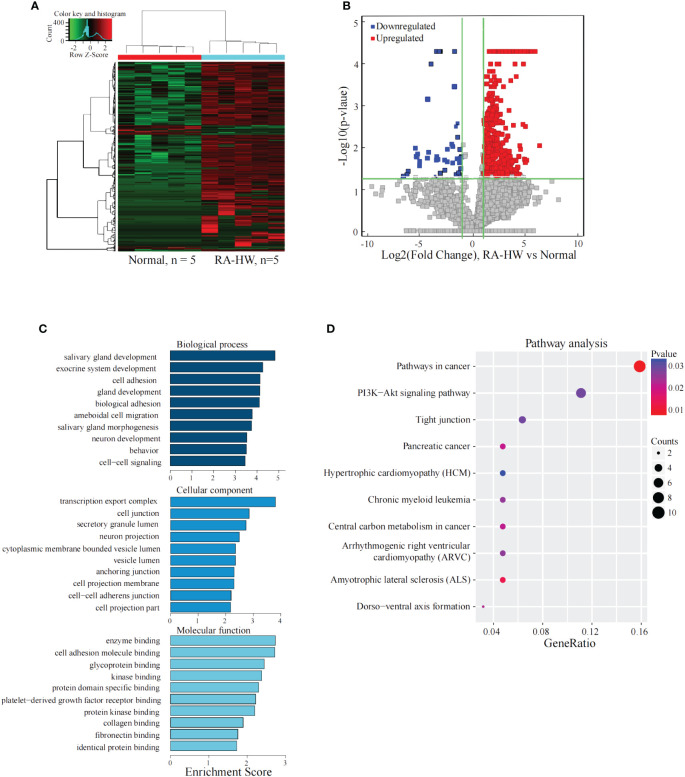
Analysis of the differential expression of the lncRNAs between the RA-HW and Normal groups. **(A)** The differential expression cluster diagram of the lncRNAs, where “red” signifies higher relative expression, and “green” indicates lower relative expression. **(B)** A volcano map depicting the differential expression of lncRNAs, with red dots representing upregulated and blue dots representing lncRNAs exhibiting a statistical significance of two times the logarithmic fold change. **(C)** Top 10 entries for GO in biological processes (GO-BP), Molecular Functions (GO-MF), and Cellular Components (GO-CC) related to the differentially expressed lncRNAs between the RA-HW and Normal groups. **(D)** A bubble diagram portraying the KEGG pathway enrichment analysis of the differentially expressed lncRNAs. The ordinate represents the pathways acquired through enrichment analysis, while the abscissa signifies the enrichment fraction. The bubble size correlates with the number of enriched genes, and the bubble color corresponds to the p-value. The intensity of the color indicates the extent of difference, with a redder hue signifying a greater difference. Additionally, the size of the circles denotes the strength of the association between the genes and pathways.

The results shown in **Figures 1A, B** indicate a total of 1103 significantly differentially expressed lncRNAs between the two groups. Compared to the Normal group, 995 lncRNAs were significantly upregulated, and 108 were significantly downregulated in the RA-HW group.

The analysis revealed significant enrichment of genes stemming from differentially expressed lncRNAs across various GO terms (**Figure 1C**). These terms included cell-cell signaling, cell adhesion, transcription export complex, cell-adhesion molecule binding, and platelet-derived growth factor receptor binding etc. The KEGG pathway enrichment analysis results for differentially expressed lncRNAs target genes between the RA-HW and Normal groups demonstrated primary enrichment of upregulated lncRNAs in pathways, such as the PI3K-Akt signaling pathway (**Figure 1D**).

### 3.2 Analysis of the differentially expressed lncRNAs between the RA-L group and normal group

**Figure 2:**
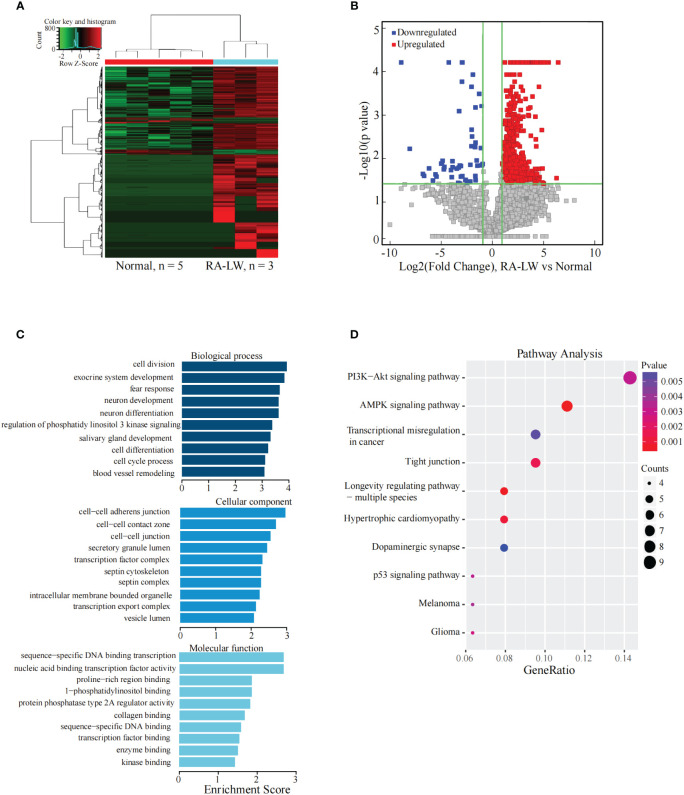
Analysis of differential expression of the lncRNAs between the RA-L and Normal groups. **(A)** The differential expression cluster diagram of the lncRNAs, where “red” signifies higher relative expression, and “green” indicates lower relative expression. **(B)** A volcano map depicting the differential expression of the lncRNAs. **(C)** Top 10 entries for GO-BP, GO-CC, and GO-MF related to differentially expressed lncRNAs between the RA-L and Normal groups. **(D)** A bubble diagram portraying the KEGG pathway enrichment analysis of the differentially expressed lncRNAs.

The results indicated 1049 significantly differentially expressed lncRNAs between the two groups. Compared with the Normal group, there were 988 significantly upregulated lncRNAs and 61 significantly downregulated lncRNAs in the RA-L group (**Figures 2A, B**).

The analysis revealed significant enrichment of genes stemming from differentially expressed lncRNAs across various GO terms (**Figure 2C**). These terms included biological adhesion, cell adhesion, cell-cell junction, beta-amyloid binding, and cell adhesion molecule binding etc. The KEGG pathway enrichment analysis results for differentially expressed lncRNA target genes between the RA-L and Normal groups demonstrated primary enrichment of upregulated lncRNAs in pathways such as hypertrophic cardiomyopathy and the AMPK signaling pathway (**Figure 2D** **).**

### 3.3 Analysis of the differentially expressed lncRNAs between the RA-LW group and normal group

**Figure 3:**
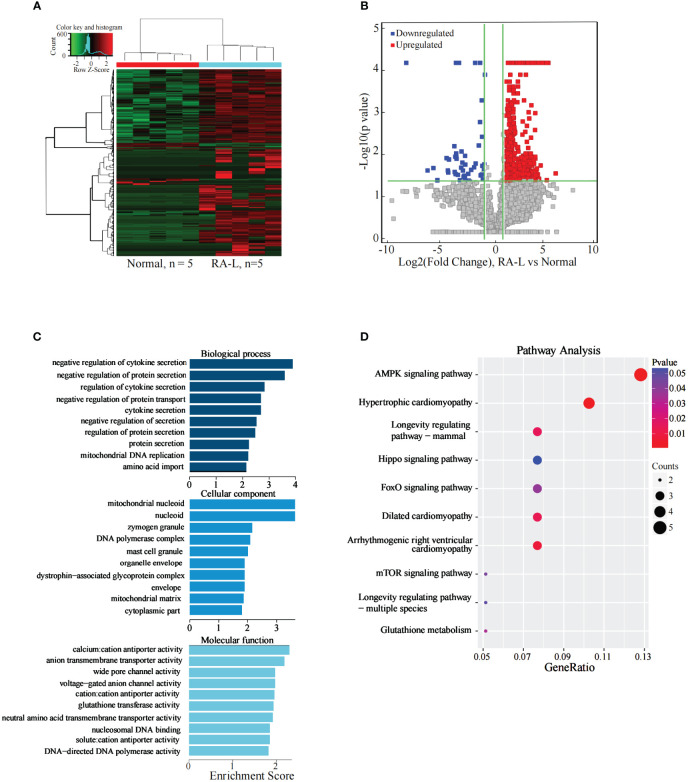
Analysis of differential expression of the lncRNAs between the RA-LW and Normal Groups. **(A)** The differential expression cluster diagram of lncRNAs, where “red” signifies higher relative expression, and “green” indicates lower relative expression. **(B)** A volcano map depicting the differential expression of lncRNAs. **(C)** Top 10 entries for GO-BP, GO-CC, and GO-MF related to the differentially expressed lncRNAs between the RA-LW and Normal groups. **(D)** A bubble diagram portraying the KEGG pathway enrichment analysis of the differentially expressed lncRNAs.

The results indicated 1350 significantly differentially expressed lncRNAs between the two groups. Compared with the Normal group, 1267 lncRNAs were significantly upregulated, and 83 were significantly downregulated in the RA-LW group (**Figures 3A, B**).

The analysis revealed significant enrichment of genes stemming from differentially expressed lncRNAs across various GO terms (**Figure 3C**). These terms included cell division, cell cycle process, cell-cell junction, transcription factor binding, and sequence-specific DNA-binding transcription factor activity etc. The KEGG pathway enrichment analysis results for differentially expressed lncRNA target genes between the RA-LW and Normal groups demonstrated primary enrichment of upregulated lncRNAs in pathways, such as the PI3K-Akt and AMPK signaling pathways (**Figure 3D**).

### 3.4 Analysis of the differentially expressed lncRNAs between the RA-HW group and RA-L group

**Figure 4:**
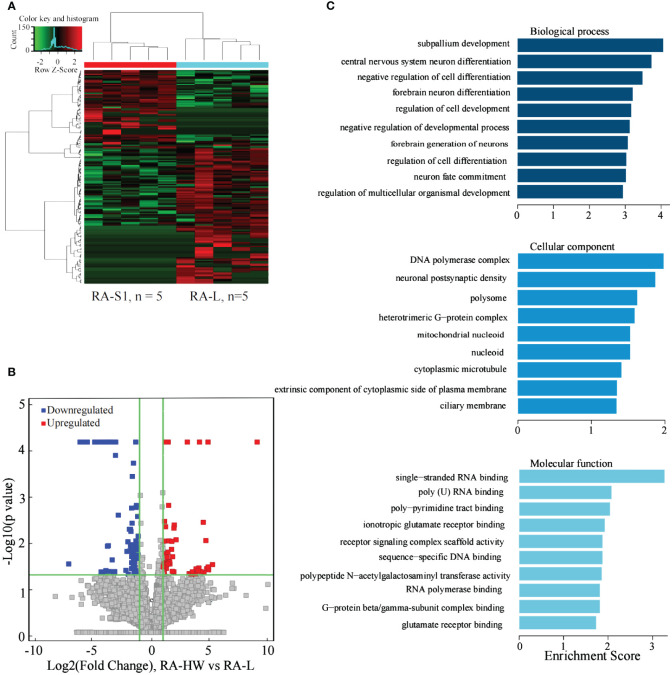
Analysis of differential expression of the lncRNAs between the RA-HW and RA-L Groups. **(A)** The differential expression cluster diagram of the lncRNAs, where “red” signifies higher relative expression, and “green” indicates lower relative expression. **(B)** A volcano map depicting the differential expression of the lncRNAs. **(C)** The top 10 entries for GO-BP, GO-CC, and GO-MF related to differentially expressed lncRNAs between the RA-HW and RA-L groups.

The results indicated that 208 lncRNAs were significantly differentially expressed between the two groups. Compared to the RA-L group, 89 lncRNAs were significantly upregulated, and 119 were significantly downregulated in the RA-HW group (**Figures 4A, B**).

The analysis revealed a substantial enrichment of genes originating from differentially expressed lncRNAs in variousGO terms (**Figure 4C**). Noteworthy terms included negative regulation of cell differentiation, regulation of cell development, polymerase complex, and single-stranded RNA binding etc.

### 3.5 Analysis of the differentially expressed lncRNAs between the RA-HW group and RA-LW group

**Figure 5:**
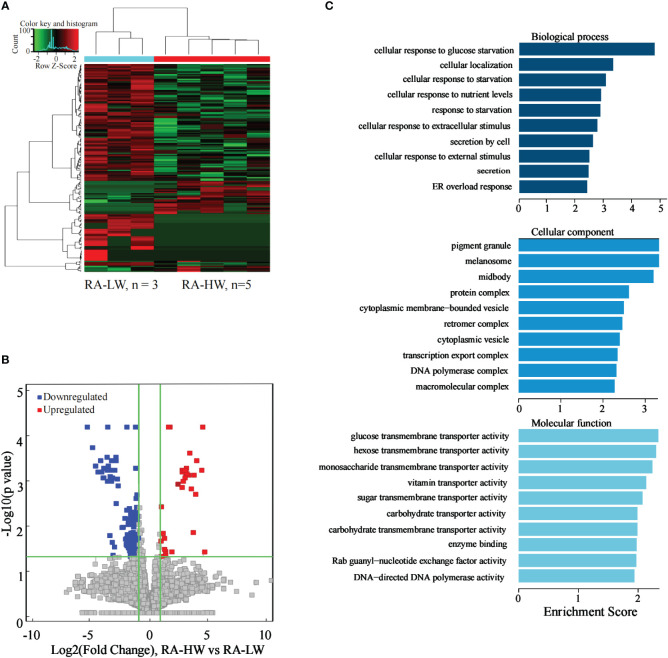
Differential expression of the lncRNAs between the RA-HW and RA-LW groups. **(A)** The differential expression cluster diagram of the lncRNAs, where “red” signifies higher relative expression, and “green” indicates lower relative expression. **(B)** A volcano map depicting the differential expression of the lncRNAs. **(C)** Top 10 entries for GO-BP, GO-CC, and GO-MF related to the differentially expressed lncRNAs between the RA-HW and RA-LW groups.

The results indicated 218 significantly differentially expressed lncRNAs between the two groups. Compared to the RA-LW group, 41 lncRNAs were significantly upregulated, and 177 were significantly downregulated in the RA-HW group (**Figures 5A, B**).

The analysis revealed a substantial enrichment of genes originating from differentially expressed lncRNAs in variousGO terms (**Figure 5C**). Noteworthy terms included cellular localization, secretion by cell, transcription, export of complex proteins, and molecular function etc.

### 3.6 Analysis of the differentially expressed lncRNAs between the RA-L and RA-LW group

**Figure 6:**
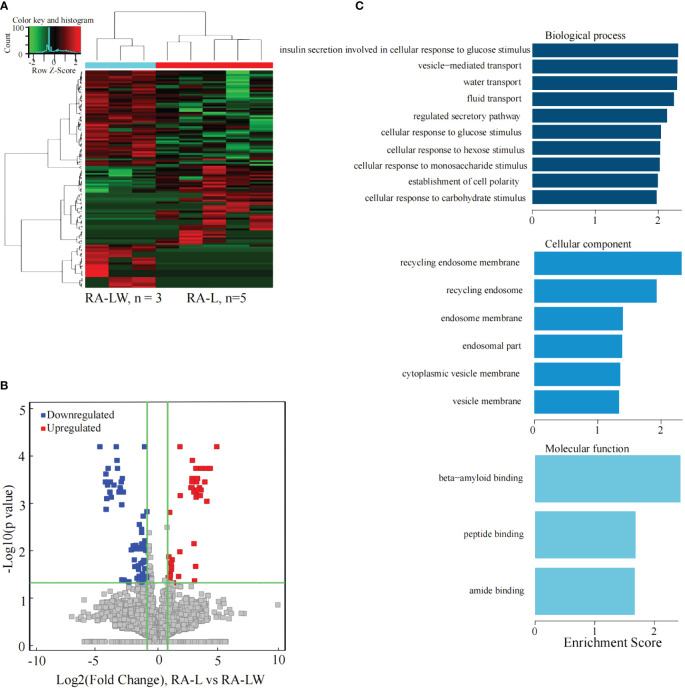
Differential expression of the lncRNAs between the RA-L and A-LW groups. **(A)** The differential expression cluster diagram of lncRNAs, where “red” signifies higher relative expression, and “green” indicates lower relative expression. **(B)** A volcano map depicting the differential expression of lncRNAs, with red dots representing upregulated lncRNAs exhibiting a statistical significance of two times the logarithmic fold change. **(C)** The top 10 entries for GO-BP, GO-CC, and GO-MF related to the differentially expressed lncRNAs between the RA-HW and RA-LW groups.

The results indicated 153 significantly differentially expressed lncRNAs between the two groups. Compared to the RA-LW group, 60 lncRNAs were significantly upregulated, and 93 were significantly downregulated in the RA-L group (**Figures 6A, B**).

The analysis revealed a substantial enrichment of genes originating from differentially expressed lncRNAs in various GO terms (**Figure 6C**). Noteworthy terms included biological process, cellular component, molecular function, and beta-amyloid binding.

### 3.7 Identification and validation for two specific lncRNAs

**Figure 7:**
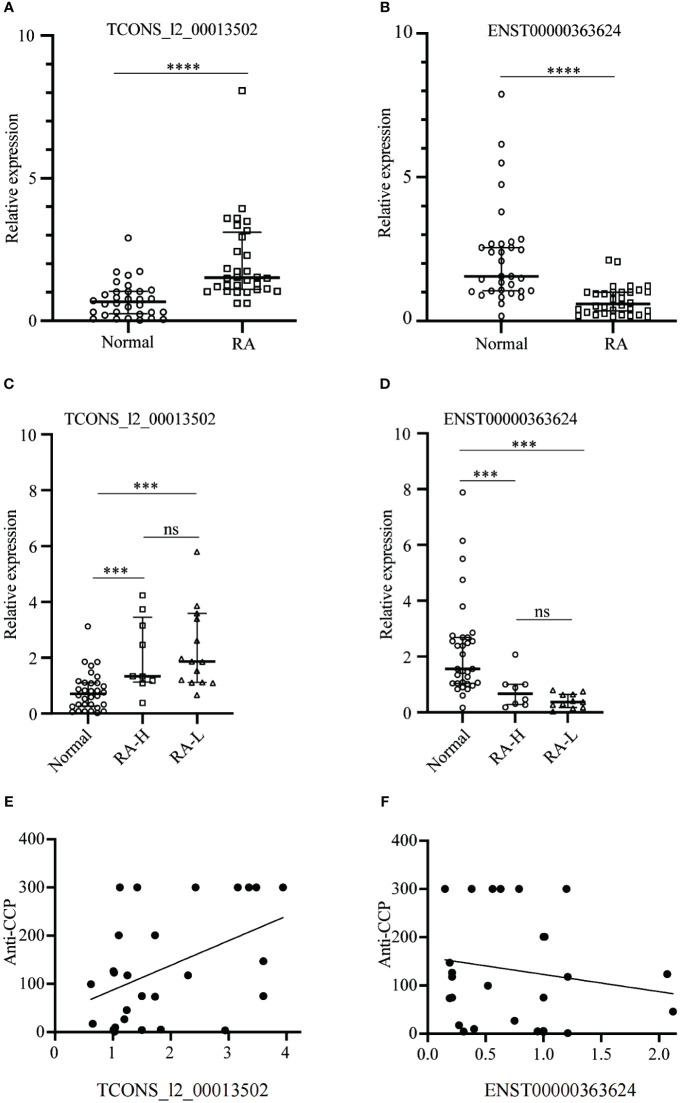
Identification and validation of the two specific lncRNAs between the RA and Normal Groups. **(A, B)** The expression of TCONS_I2_00013502 **(A)** and ENST00000363624 **(B)** in the serum exosomes of patients with RA compared to Normal. **(C, D)** The expressions of TCONS_I2_00013502 **(C)** and ENST00000363624 **(D)** across different groups of patients with RA. **(E, F)** Correlation analysis of serum exosomal TCONS_I2_00013502 **(E)** and ENST0000036362 **(F)** with the anti-CCP antibody levels of patients with rheumatoid arthritis. ***P < 0.001, ****P < 0.0001, ns, no significance.

The RT-qPCR results demonstrated significant differential expression of TCONS_I2_00013502 and ENST00000363624 among the 18 candidate lncRNAs (*p* < 0.05). The differential expression of lncRNAs between the RA group and the Normal group had a statistical significance set at *p* < 0.05. In comparison to the Normal group, the expression of TCONS_I2_00013502 in the serum exosomes of patients with RA was significantly elevated (*p* < 0.05) (**Figure 7A**), whereas the expression of ENST00000363624 exhibited a significant decrease in the serum exosomes of patients with RA (*p* < 0.05) (**Figure 7B**).

Based on the sequencing results, we observed similar expression levels of these two distinct RNAs in the RA-HW and RA-LW groups, exceeding those in the normal group by more than three-fold. In contrast, the RA-L group displayed expression levels that were twice as high as those in the normal group. Consequently, we grouped patients from the RA-HW and RA-LW groups together as the RA-H group for subsequent analyses and compared them with the Normal and RA-L groups. Statistical analysis revealed significant differences between the Normal group and both the RA-H and RA-L groups (*p* < 0.05),however, no significant differences were observed between the RA-H and RA-L groups (*p* > 0.05) (**Figures 7C, D**).

The correlation analysis of TCONS_I2_00013502 and ENST00000363624 with anti-CCP antibody levels revealed that TCONS_I2_00013502 (r = 0.45) was significantly and positively correlated with anti-CCP antibody levels (*p* < 0.05). Conversely, ENST00000363624 (r = -0.16) showed no significant correlation with anti-CCP antibody levels (*p* > 0.05) (**Figures 7E, F**).

### 3.8 ROC curve analysis

**Figure 8:**
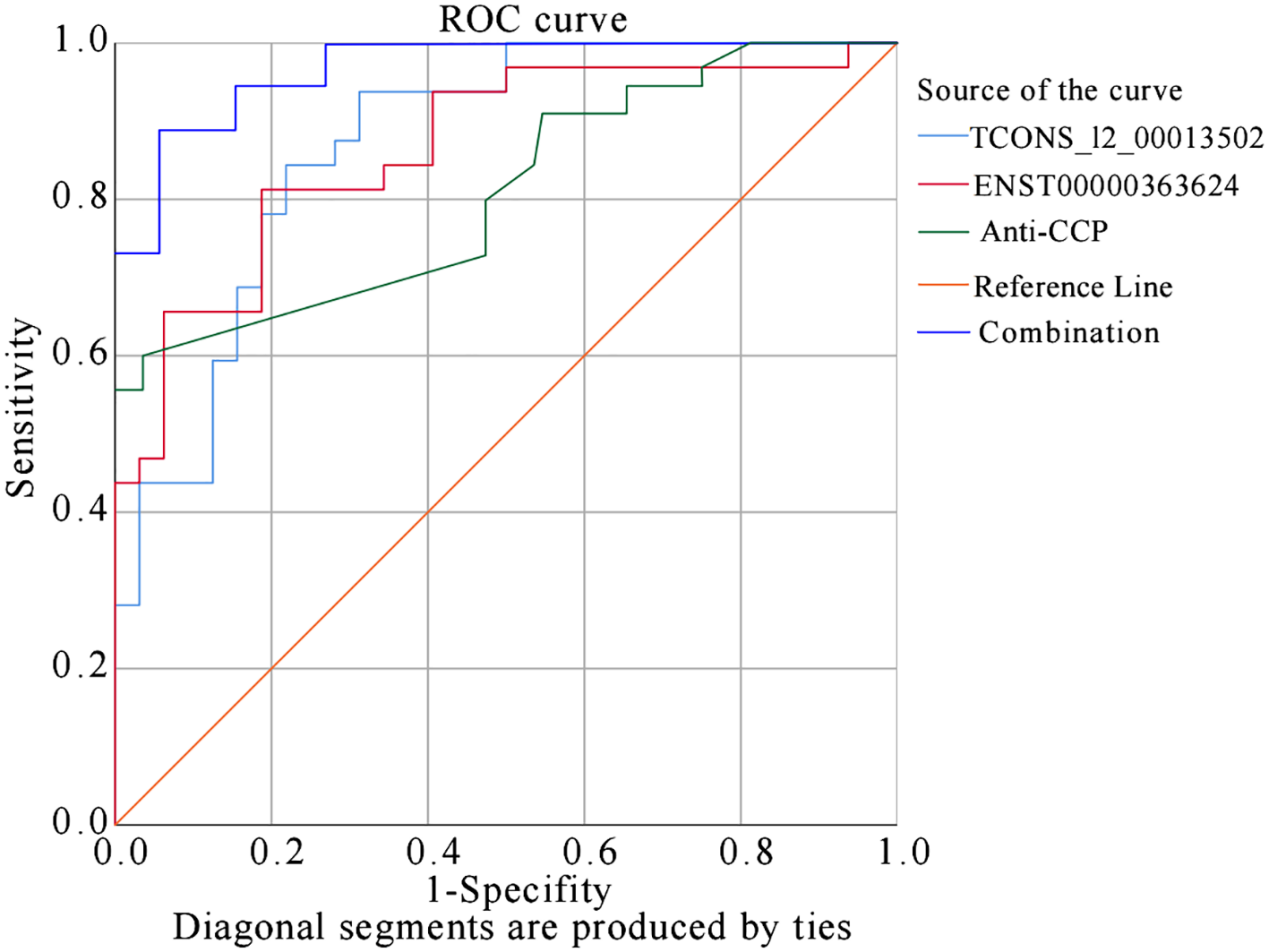
ROC Curve Analysis for TCONS_I2_00013502 and ENST00000363624 in predicting RA.

Based on predicted probabilities and true values, the ROC curve had area under the curve (AUC) values of 0.870 for TCONS_I2_00013502, 0.864 for ENST00000363624, and 0.782 for anti-CCP. The joint index had an AUC of 0.966, indicating strong predictive capabilities for TCONS_I2_00013502, ENST00000363624, and anti-CCP in relation to RA. When evaluating TCONS_I2_00013502 separately, it achieved the highest Youden index of 0.625 (sensitivity: 93%, specificity: 68.7%). ENST00000363624 achieved the highest Youden index of 0.594 (sensitivity: 81.3%, specificity: 78.1%). Additionally, anti-CCP displayed the highest Youden index of 0.48 (sensitivity: 78%, specificity: 82.1%). However, considering the combined sensitivity and specificity of TCONS_I2_00013502, ENST00000363624, and anti-CCP, the highest Youden index was 0.76 (sensitivity: 92%; specificity: 84%). This demonstrates improved discriminatory power for RA prediction when all three indicators are used together (**Figure 8**).

## 4 Discussion

RA is a systemic, chronic inflammatory disease characterized by progressive joint destruction, deformity, and disability caused by abnormal immune system activity. In recent years, increasing evidence has suggested that lncRNAs play a crucial role in autoimmune diseases, including RA, serving not only as biomarkers for disease diagnosis and prognosis but also as potential therapeutic targets in mechanistic studies (17, 18). Exosomes are nanosized extracellular vesicles secreted by cells that carry biologically active substances, such as nucleic acids, proteins, and lipids (19). They play roles in various physiological and pathological processes in the body. Previous studies have shown that the lncRNAs in serum exosomes are crucial regulatory molecules in the pathogenesis and progression of RA (20–22).

Increasing evidences suggest a significant association between lncRNA abnormalities and the occurrence and development of RA. In previous studies, two lncRNAs, lnc-AL928768.3 and lnc-AC091493.1, found in the synovial tissue of patients with RA, were positively correlated with the Disease Activity Score in 28 joints (DAS28) and C-reactive protein levels, making them potential diagnostic and activity indicators for RA (23, 24). Furthermore, the lncRNA, PICSAR, is highly expressed in fibroblast-like synovial (FLS) cells and the synovial fluid of patients with RA, promoting FLS cell proliferation, migration, and invasion. The inhibition of PICSAR expression significantly reduces the production of IL-6, IL-8, and MMP-3 (25). Studies have also found that exosomes derived from peripheral blood mononuclear cells (PBMCs) can play a crucial role in the development of RA by regulating the miR-23a/MDM2/SIRT6 axis through the transmission of lncRNA NEAT1 (26). These lncRNAs and their downstream targets are potential targets for the diagnosis and treatment of RA. Furthermore, some lncRNAs, such as HOTAIR and GAS5, have been used clinically for the diagnosis and treatment of RA (27).

In this study, RNA sequencing identified 3,210 upregulated and 252 downregulated lncRNAs in the peripheral serum exosomes of the three RA patient subgroups compared to those in the normal group. GO and KEGG enrichment analyses indicated that these lncRNAs are associated with various immune responses and inflammatory diseases. Notably, TCONS_00026389, TCONS_00028428, TCONS_l2_00013502, uc010wia.1, TCONS_l2_00003048, TCONS_00028193, TCONS_00028189, NR_033191, TCONS_00028422, and TCONS_00028426 were identified as commonly upregulated lncRNAs. ENST00000363624, ENST00000365328, ENST00000363444, ENST00000363618, ENST00000458748, ENST00000365312, ENST00000391267, and ENST00000437681 were commonly downregulated across the three RA patient subgroups. In the RA-HW group, there were 89 upregulated and 119 downregulated lncRNAs compared to those in the RA-L group. Additionally, 41 lncRNAs were upregulated, and 177 were downregulated compared with those in the RA-LW group. The RA-LW group showed 60 upregulated and 93 downregulated lncRNAs compared to the RA-L group. Subsequently, we verified the differential expression of RNA among these groups, which is important for studying RA disease activity. Among the different groups, we only enriched GO analysis but not KEGG analysis. The enrichment of GO analysis exclusively, without concurrent enrichment in KEGG analysis, might imply a narrower impact of differentially expressed lncRNAs on the pathways. This observation indicates a more targeted influence on specific biological processes rather than a broader effect across various pathways influenced by these lncRNAs.

As validation, TCONS_I2_00013502 levels were significantly elevated, and ENST00000363624 levels were significantly decreased in the peripheral serum exosomes of patients with RA compared to those in the normal group (*p* < 0.05). The ROC curve analysis suggested that TCONS_I2_00013502 and ENST00000363624 could serve as novel biomarkers for diagnosing RA, with the sensitivity and specificity values provided. The combination of TCONS_I2_00013502 and ENST00000363624 exhibited high sensitivity and specificity for the diagnosis of RA. The enrichment of these dysregulated lncRNAs in pathways associated with key cellular processes, such as the salivary gland and the transcription export complex, as well as pathways like PI3K-Akt signaling, and those pathways related to cancer, implies that those lncRNAs are involved in RA pathophysiology. The diverse functional enrichment of lncRNAs emphasizes their multifaceted roles in modulating the biological pathways linked to RA pathogenesis. The PI3K-Akt signaling pathway plays a critical role in various cellular processes, including cell survival, proliferation, and immune response. Dysregulation of this pathway has been associated with the onset and progression of RA (28, 29).

In our study, we utilized two major databases, miRDB and TargetScan to predict downstream genes regulated by these significantly differentially expressed lncRNAs. We also conducted pathway prediction on the identified downstream genes. Our findings revealed that the downstream miRNAs were mainly enriched in the PI3K-Akt and MAPK signaling pathways. MAPK signaling is also crucial cascades involved in numerous cellular processes, such as cell growth, proliferation, differentiation, and inflammatory responses (30, 31). Dysregulation of these pathways has been implicated in the development and progression of RA. Previous studies have suggested that several dysregulated lncRNAs may influence the expression or activity of key genes or proteins within these pathways (28, 32), thereby altering cellular processes and contributing to RA pathogenesis. Therefore, our future research will focus on investigating the specific roles of these lncRNAs within the inflammatory pathways. Identifying specific dysregulated lncRNAs within these pathways, along with their target genes or proteins, could potentially lead to the identification of therapeutic targets for RA. Targeting these lncRNAs or the molecules they regulate may provide novel strategies for intervening in or treating this complex autoimmune disease.

The correlation analysis results revealed distinct differences between TCONS_I2_00013502 and ENST00000363624 with respect to the anti-CCP antibody levels. Specifically, TCONS_I2_00013502 exhibited a significant positive correlation with the anti-CCP antibody levels, suggesting a close association with the generation or regulation of these antibodies. In contrast, ENST00000363624 exhibited a relatively weak and non-significant correlation, indicating a limited impact on anti-CCP antibody levels.

These findings offer insights for further investigation into the roles of these genes in RA. The positive correlation of TCONS_I2_00013502 suggests a potential regulatory role in immune responses, warranting additional functional studies to elucidate its specific mechanisms. Conversely, the weak correlation with ENST00000363624 may necessitate a more thorough investigation to determine its role in specific physiological and pathological conditions. Therefore, our analysis results will guide future in-depth studies on the potential roles of these genes in autoimmune diseases, underscoring the need for further exploration to understand the mechanisms underlying anti-CCP antibody generation.

Although this study successfully identified potential lncRNA biomarkers in the serum exosomes of RA patients, it had some limitations. The study did not explore the tissues or cells from which the serum exosomal lncRNAs originated. This limitation may partly stem from the relatively small sample size, emphasizing the need for validation in a larger RA population. Additionally, the study overlooked the differential diagnosis of other RA-related conditions, such as gout and osteoarthritis. Future studies will prioritize expanding the sample size, refining the differential diagnosis, and conducting cell experiments. These steps would aim to deepen our comprehension of the upstream and downstream mechanisms involved in the regulation of TCONS_I2_00013502 and ENST00000363624 in RA pathogenesis. The absence of differential expression of these genes across various groups may be due to the limited number of samples or potentially insufficient categorization. This aspect calls for further experiments to confirm our results, thus representing a notable limitation of this study.

In conclusion, our study provides comprehensive insights into the differential expression patterns of lncRNAs in peripheral serum exosomes of RA patients. These findings highlight the potential importance of specific lncRNAs in immune responses and inflammatory diseases, as well as their potential as diagnostic biomarkers for RA. These findings hold promise for advancing early and accurate diagnosis, thereby facilitating prompt therapeutic interventions and improving patient outcomes in RA.

## Data availability statement

The data presented in the study are deposited in the GEO database, accession number GSE271161.

## Ethics statement

The studies involving humans were approved by the Affiliated Hospital of Guangdong Medical University (PJ2014072). The studies were conducted in accordance with the local legislation and institutional requirements. The participants provided their written informed consent to participate in this study. Written informed consent was obtained from the individual(s) for the publication of any potentially identifiable images or data included in this article.

## Author contributions

HW: Data curation, Methodology, Writing – original draft. QC: Data curation, Methodology, Writing – original draft. SW: Conceptualization, Data curation, Writing – original draft. CY: Data curation, Writing – review & editing. LX: Conceptualization, Data curation, Resources, Writing – original draft. HX: Data curation, Investigation, Resources, Writing – original draft. TX: Data curation, Funding acquisition, Writing – review & editing. QP: Funding acquisition, Investigation, Methodology, Supervision, Writing – review & editing.
